# miRNA and antisense oligonucleotide-based α-synuclein targeting as disease-modifying therapeutics in Parkinson’s disease

**DOI:** 10.3389/fphar.2022.1034072

**Published:** 2022-11-15

**Authors:** Vasanti Suvarna, Kajal Deshmukh, Manikanta Murahari

**Affiliations:** ^1^ Department of Quality Assurance, SVKM’s Dr. Bhanuben Nanavati College of Pharmacy, Mumbai, India; ^2^ Department of Pharmacy, Koneru Lakshmaiah Education Foundation, Vaddeswaram, AP, India

**Keywords:** Parkinson’s disease, alpha-synuclein, antisense oligonucleotides, miRNA, neurodegenerative diseases

## Abstract

α-synuclein is the synaptic protein majorly involved in neuronal dysfunction and death and it is well known for the last two decades as a hallmark of Parkinson’s disease. Alpha-synuclein is involved in neurodegeneration mediated through various neurotoxic pathways, majorly including autophagy or lysosomal dysregulation, mitochondrial disruption, synaptic dysfunction, and oxidative stress. Moreover, the alpha-synuclein aggregation has been associated with the development of several neurodegenerative conditions such as various forms of Parkinson’s disease. The recent discovery in oligonucleotide chemistry has developed potential alpha-synuclein targeting molecules for the treatment of neurodegenerative diseases. The present review article focuses on recent advances in the applications of oligonucleotides acting *via* alpha-synuclein targeting mechanisms and their implication in combating Parkinson’s disease. Moreover, the article emphasizes the potential of miRNAs, and antisense oligonucleotides and the challenges associated with their use in the therapeutical management of Parkinson’s disease.

## 1 Introduction

Parkinson’s disease (PD) is the second most globally prevalent progressive nervous system linked movement disorder after Alzheimer’s disease (Srivastava et al., 2011). Presently, more than ten million of the global population is suffering from PD. Age is a crucial risk element in the progression of PD with its incidence increasing with age, and an estimated four percent of PD patients were diagnosed below 50 years of age, and about 1–2% of the over-60 population was reported to suffer from the disease. The average age of PD manifestation is around 60 years, which might range from as early as 20 years to over 90 years depending on the causes involved (Latourelle et al., 2009). PD is characterized by non-motor clinical symptoms like constipation, cognitive debility, bladder dysfunction, orthostatic hypotension; REM sleep behavior disorder; sleep issues, dementia, pain, depression, and olfactory impairment, with motor symptoms like tremor, rigidity, bradykinesia, gait and balance abnormalities, and postural instability (Doxakis 2020). PD being a multi-factorial disease involves an intricate interplay of multiple genes with their effects, modified by susceptibility alleles, environmental exposures, and gene-environment interactions; all together impacting the developing and aging brain directly. As PD symptoms develop considerably later than the actual onset of neuron degeneration, most patients remain untreated until the symptoms appear (Dorsey et al., 2018).

In surviving neurons, proteinaceous cytosolic aggregates known as Lewy bodies (LB) and thread-like fibrils in cellular processes known as Lewy neurites (LN) are the putative hallmarks of PD. α-Synuclein (α-syn) is a small peripheral membrane protein encoded by the SNCA gene mainly localized into the axon terminal of neurons. It preferentially deposits in Lewy bodies present in the cytoplasmic space of dopaminergic neurons of the substantia nigra pars compacta region of the brain, resulting in cell death and reduction in dopamine levels (Cheruvara et al., 2015). Therefore, inhibition of α-syn levels by suppressing the expression of the SNCA gene encoding α-syn could be of significant importance to slow PD progression. The LB formation causes neurotoxicity including fibrillation, post translational changes, and interacts with membrane organelles, interrupting key cellular activities and triggering synaptic dysfunction and mitochondrial toxicity (Mahul-Mellier et al., 2020). Various research findings have indicated the involvement of alpha-synuclein-(PARK 1,2,3,4,5,6, and 7) proteins in the pathogenesis of PD. PARK4 is an autosomal dominantly inherited PD resulting due to multiplication such as duplication or triplication of the SNCA gene including cognitive impairment as a clinical feature. This results in high levels of α-syn protein consequently causing its accumulation and exhibiting neurotoxic effects. PARK4 patients exhibit an early appearance of PD symptoms with no associated pathological mutations, therefore, inhibition of α-syn aggregation could be a potential target in PD treatment (Fukusumi et al., 2021). Aggregation of α-syn induces neurodegeneration by causing a direct and potentially dangerous gain-of-function (Mahul-Mellier et al., 2020).

## 2 Alpha-synuclein (α-syn)

**FIGURE 1:**
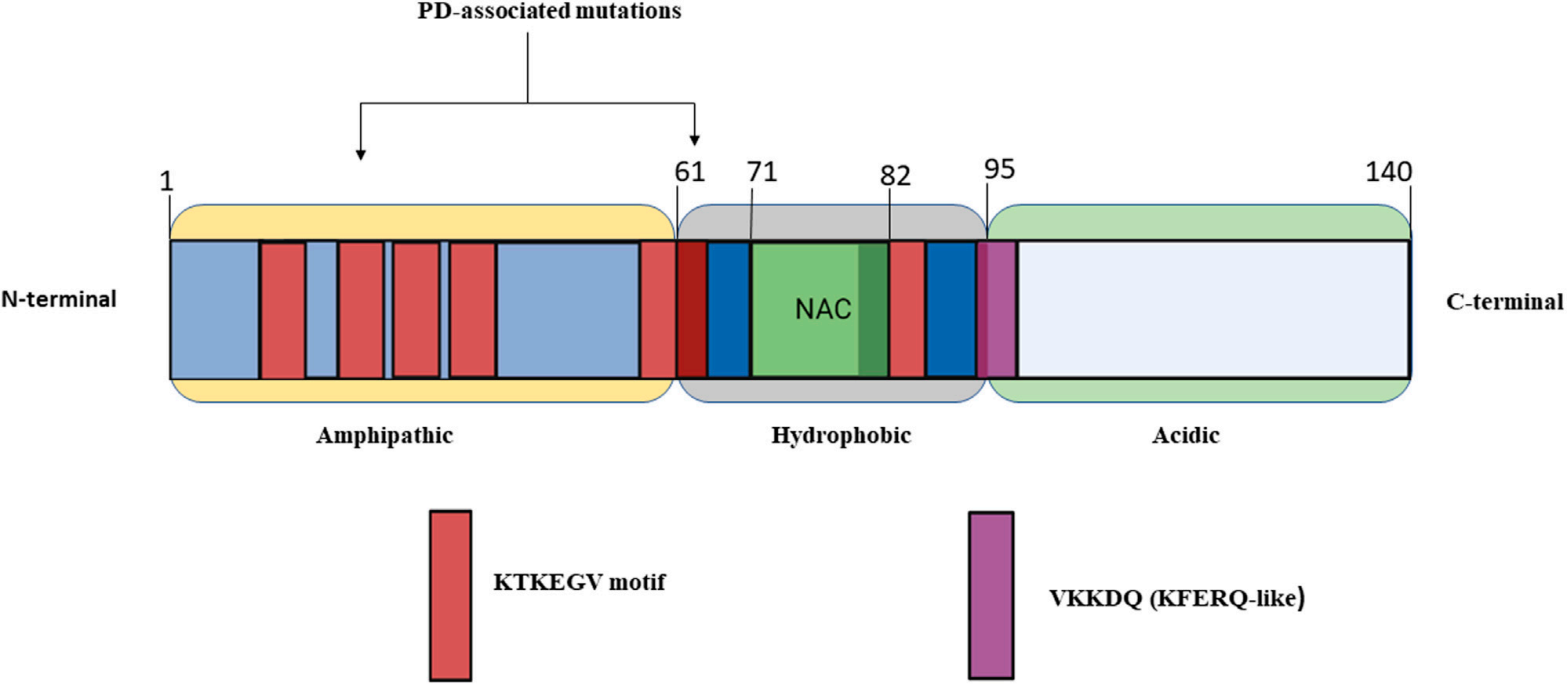
Simplified structure of alpha-synuclein made up of 140 amino acid residues and broadly categorized into three major regions of α-Syn (1) Amphipathic (2) Hydrophobic (3) Acidic.

α-syn is a member of intrinsically disordered proteins comprising of 140 amino acid residues encoded by SNCA gene (Lee and Kim 2012; Weinreb et al., 1996). The primary structure of α-syn is comprised of three parts (Figure 1): 1) The N-terminal region (residues 1–60) contains a sequence of eleven-amino acid repeats with a preserved KTKEGV motif that acquire an amphipathic α-helical conformation. Additionally, the SNCA gene exhibits familial mutations such as A30P, E46K, H50Q, G51D, A53E, A53V, and A53T found in this region. 2) The central hydrophobic amyloidogenic NAC (non-amyloid-component) region is comprised of residues (61–95) and two more KTKEGV repeats, both implicated in the development of amyloid fibrils. This region can transit from a randomly coiled state to a strongly hydrophobic and easily aggregating beta-sheet form. 3) The polar C-terminal region (residues 96–140) is extremely acidic and contributes to the total net negative charge. This region serves as a site for the majority of post-translational modifications and mediates interactions of α-syn with other proteins, ligands, and metal ions. The ablation of this region is observed to enhance α-syn aggregation and abolish its chaperone-like action. In addition, truncation of the C-terminal region causes an elevation in filament assembly. During the assembly of recombinantly expressed α-syn, conformational changes occur to achieve the cross-β structural characteristic of amyloid. The different isoforms of α-syn include β-synuclein (β-syn) and γ-synuclein (γ-syn). Moreover, β-syn lack a NAC region at the N-terminus and possesses higher tendency to attain helical conformations than α-syn. These attributes may confer its stable native unfolded state by blocking oligomerization. On other hand β-syn was observed to exhibit on neurotoxic effect against cultured neuronal cells and caused progressive neurodegeneration. Slight change in Ph induces β-syn fibrillation (Mehra et al.,2019; Guerrero-ferreira et al., 2020; Min et al., 2020).

### 2.1 Mechanism of alpha-synuclein aggregation

**FIGURE 2:**
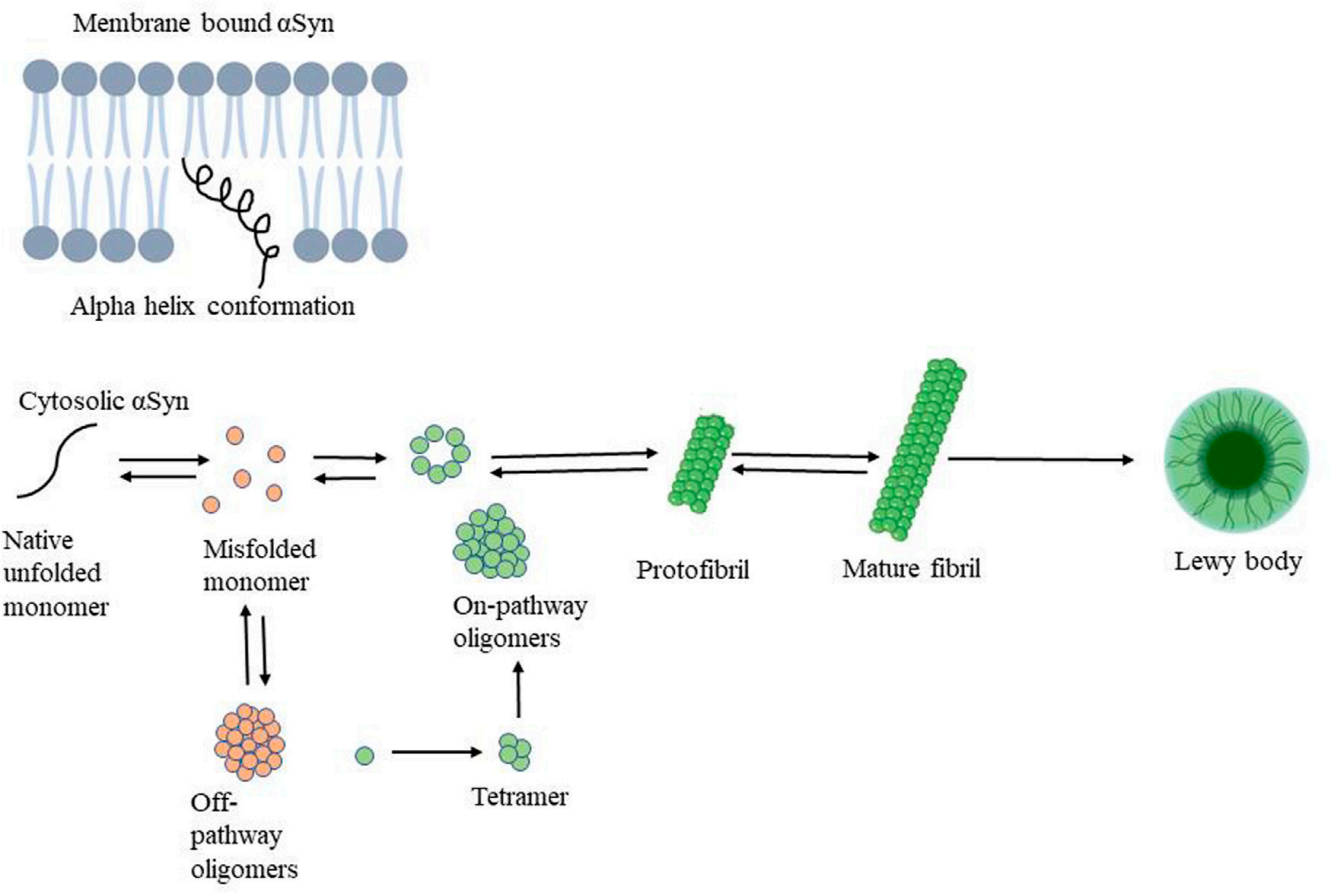
Mechanism of alpha-synuclein aggregation followed by oligomer, protofibril, mature fibril formation resulting in Lewy body responsible for PD pathogenesis.

Many proteins linked to neurodegenerative disorders adopt a pathological conformation, inducing similar conformational changes in endogenous proteins, resulting in a self-perpetuating cycle. For example, endogenous α-syn is mainly found in neurons, where it is particularly abundant at the presynaptic terminal of neurons (Hijaz and Volpicelli-Daley 2020). The C-terminal region seems to play a role in preventing α-syn aggregation as the C-terminal region a truncated form of α-syn (residues 1–120) was more susceptible to form filaments in comparison to full-length protein (Sorrentino et al., 2018) α-syn’s typical conformation is either a disordered monomer or a multimeric alpha-helical conformation. α-syn folds into two curved antiparallel helixes joined by a linker made up of amino acids 38–44. Thus, forming helical tetramer and higher-order oligomers, mostly upon interaction with membrane vesicles. Although α-syn appears to naturally bind micelles, the ratio of the two components is critical. Once the threshold is surpassed the α-syn starts condensing on the micelle and initiates oligomerization. During *in vitro* oligomerization, α-syn’s structure shifts to a beta-sheet-rich conformation, resulting in aggregates with amyloid properties. Here, protofilaments are generated first, followed by oligomers and protofibrils, which combine to form amyloid fibrils (Figure 2) (Xylaki and Fleming 2019; Cascella et al., 2022). In PD brains, filaments of α-syn in the amyloid conformation distinguish Lewy bodies from Lewy neurites. Soluble α-syn undergoes conformational alteration and assembles into β-sheet under pathological circumstances, such as exogenous exposure of preformed fibrils (Hastings et al., 2022). Although it is well established that misfolded α-syn can spread and propagate in a prion-like manner it is important to note that other regional or cell-autonomous factors that are likely to make neurons vulnerable to damage, which is what causes the neurotoxicity seen in Parkinson’s disease (Surmeier et al., 2017). For example, In the intestine, α-synuclein exists in a monomeric form and can be released as free protein or in the form of exosomes from neurons into the paracellular space. It may then invade neighboring neurons *via* endocytosis. In experimental models of PD, it was observed that only neurons with intact synaptic connections possess invasion of α-syn from neuron to neuron in a receding manner from enteric nerves to the vagus nerve (Liddle 2018). Presently, the mechanisms behind the production of intracytoplasmic inclusions (α-syn) in neuronal cells are not fully understood. One probable cause could be poor autophagic machinery, which is unable to deal with the high intracellular concentration of α-syn. Several investigations have found that α-syn aggregation is caused by decreased autophagy (Fellner et al., 2021). Moreover, mitochondrial dysfunction, oxidative stress, ubiquitin-proteosome system, endoplasmic reticulum stress and unfolded protein response are also responsible for PD pathogenesis (Bortolozzi et al., 2021). Toxicity of varying degrees is caused by both α-syn fibrils and oligomers (Hijaz and Volpicelli-Daley 2020). Both the cytosol and the extracellular interstitial fluid contain α-syn. The oligomeric species were discovered in the cerebrospinal fluid of PD patients, allowing seeding and aggregation of monomeric α-syn (Cascella et al., 2022). Oligomers can be classified as follows: toxic “on-pathway” or non-toxic “off-pathway” to amyloid fibril formation. The extent of internal β-sheet structure and the exposure to hydrophobic surfaces are likely associated with toxicity. In contrast to non-toxic oligomers, toxic oligomers are often β-rich and exposed to more hydrophobic residues, allowing them to penetrate the membrane. However, other findings suggest that fibrils, as compared to oligomers, may play a larger role in toxicity mechanisms. As evidenced by ongoing research into the impact of α-syn in PD pathogenesis, the focus has shifted from protein inclusions to oligomers. Protein inclusions in PD and other neurodegenerative disorders are not the sole source of neurotoxic proteins. Since oligomers have been recognized as important contributors to toxicity, their involvement in neurodegeneration should be further explored. Because toxic α-syn oligomers are found in the extracellular space and their role in the early pathogenesis of PD, generating ligands for oligomeric α-syn rather than intracellular fibrillar α-syn may be more practical and clinically beneficial (Kalia et al., 2013).

### 2.2 Signaling pathways for ASO

The majority of ASOs developed are designed to possess a common pathway for degradation of the targeted RNA such as by either attracting RNase H1 or by anchoring to and activating argonaute 2 (Ago2) (Table 1). RNase H1 is an endogenous nuclease, encouraging the cleavage of the RNA in an RNA-DNA heteroduplex (Bennett 2019). ASOs that target the RNA interference pathway, such as siRNAs, typically enter cells as a modified RNA, or RNA duplex, where two strands get separated inside the cell and the antisense strand (also known as the guide RNA) adheres to Ago2 comprising of RNase H like domain resulting in degradation of RNA in RNA-Ago2 complex. The free Ago2 now is ready to target another RNA and the cycle continues (Bennett et al., 2021). Additionally, ASOs possess one more mechanism involving RNA interference pathways, where the oligonucleotides possessing RNase H1 mechanism binds to the targeted RNA first followed by the recruitment of enzyme. While this is exactly the opposite with siRNA where binding with enzyme occurs first followed by binding of the enzyme-oligonucleotide duplex to the RNA (Bennett 2019). Moreover, there are other pathways by which ASOs can trigger the degradation of the targeted RNA such as non-RNase-H1-dependent downregulation mechanisms that involve either inducing alternative splicing of pre-mRNA to produce mRNAs comprising of premature termination codons, which are nonsense-mediated mRNA decay (NMD) targets or acting on mature mRNA to induce no-go decay (Crooke et al., 2021). A mechanism that does not cause the RNA transcript to degrade but instead reduces protein synthesis is by blocking protein translation by ASOs. These mechanisms are termed non-degradative mechanisms involving modulation of pre-mRNA splicing to boost the inclusion or exclusion of exons and degradation of the RNA transcript through nonsense-mediated decay. ASOs can also be used to block or displace access to the target RNA by proteins and other RNA. ASOs can also be used to block or displace proteins and other RNA from accessing the target RNA. Last but not least, ASOs can be used to enhance protein translation by blocking the translation of upstream open reading frames (uORF), disrupting regulatory RNA structures, and blocking microRNA access to the transcript’s 3′-UTR (Bennett et al., 2021).

**TABLE 1:** Signalling pathways for oligonucleotide-based therapeutics for PD.

Oligonucleotide based therapeutics	Signaling pathways
Antisense oligonucleotides	RNase H1 mediated degradation
	Non-sense mediated decay (NMD)
	AGO2 mediated degradation
	No-go-decay
	Translational arrest
	5′ Cap inhibition
	Altering polyadenylation site
	Splicing modulation
	Inhibition of miRNA function
	Modulating uORF and TIE utilization
	RNA activation
miRNA	Target miRNA degradation
	Translational repression
	miRNA stabilization

## 3 Antisense oligonucleotides (ASO) based therapeutics for Parkinson’s disease

Synthetic nucleic acid analogs known as antisense oligonucleotides (ASOs) can be designed to anneal to particular RNA sequences (Li et al., 2020). Based on the chemical design employed and the location of the target, ASOs can modify splicing, hinder or deflect translation inception, or boost the degradation of disease-causing RNA by recruitment of endonucleases such as RNaseH1. Single-stranded ASOs first bind to their target RNA by Watson-Crick base-pairing. ASOs are designed in two different paradigms: 1) gapmers designed for gene knockdown mediated by RNase H, which binds and cleaves RNA/DNA heteroduplexes primarily in the nucleus, in this a central deoxynucleotide region is flanked at both ends by modified ribonucleotides and, 2) mixmers which carry out microRNA (miRNA) inhibition or splice modulation by serving as steric blockers of the miRNA guide strand or specific splice sites, respectively, without causing RNA degradation (Holm et al., 2022). This has caught the interest of many researchers leading to several chemical modifications providing stability to ASOs but in return, it decreases their ability to cross the cell membrane. To overcome this issue, ASOs are conjugated to molecules that have an affinity for specific cell membrane proteins, such as receptor ligands or antibodies, allowing the ASO conjugate to enter the cell *via* receptor-induced endocytosis. Thus, improves the cellular uptake of ASOs and targets specific cell types. (Grabowska-Pyrzewicz et al., 2021).

**FIGURE 3:**
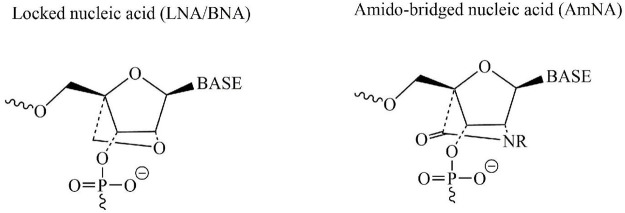
Chemical structures of two nucleotide modification called AmNA and LNA/BNA possessing a potential therapeutic candidate for treatment of PD.

Recent breakthroughs in nucleic acid modification technology have resulted in remarkable improvements in the development of nucleic acid medications with improved binding affinity, better *in vivo* stability, and reduced toxicity. Considering that excessive levels of α-synuclein protein can induce neurodegeneration in PD, a study was designed and aimed to suppress the expression of the SNCA gene responsible for the production of α-synuclein protein and ultimately prevent neurodegeneration in PARK4 patients. PARK4 is a kind of autosomal dominantly inherited PD characterized by cognitive impairment and caused by SNCA gene duplication or triplication while healthy individuals have one copy of the SNCA gene on each chromosome. This ultimately results in excessive levels of α-synuclein protein accumulating in neurons. The introduction of a novel technology of nucleotide modification called amido-bridged nucleic acid (AmNA), a locked nucleic acid (LNA/BNA) analog (Figure 3), resulting in increased target binding affinity, increased resistance to DNA-degrading enzymes, and reduced toxicity. To achieve degradation of SNCA mRNA, optimization of the nucleic acid sequence and Gapmer-ASO structure was carried out. Gapmer-ASO structure comprised of normal DNA in the center (gap) and altered nucleic acids at both ends of a linear single-stranded oligonucleotide. A Gapmer-type antisense nucleic acid with this structure coheres to its target mRNA, while the RNA-degrading enzyme RNase H can detect the DNA:RNA hybrid structure created by the DNA component of the ASOs and the mRNA target. This leads to the degradation of SNCA mRNA, which resulted in a decrease in α-synuclein protein levels and ultimately prevents neurodegeneration (Nakamori et al., 2019).

Currently, ASO-based therapeutics for the treatment of CNS disorders use an invasive administration, for example, intrathecal or intracerebroventricular administration. Systemic ASO delivery to the brain has been one of the most difficult obstacles in drug delivery and ASO therapeutics, inflicting a significant burden on patients. There are various focused studies on overcoming these challenges to provide the best possible treatment approach.

### 3.1 Multiple glucose-modified nanocarriers

Multiple glucose-modified nanocarriers that can be linked by glucose transporter-1 (GLUT1) expressed on brain capillary endothelial cells are developed for stable encapsulation of ASOs that can cross the BBB *via* active translocation of GLUT1. Thus, enabling non-invasive ASO administration into the brain by crossing BBB using glycemic control as an external trigger, and has an immediate and considerable impact on ASO treatment of CNS disorders (for example, HD and ALS), symptoms of which mainly occur in the cerebral cortex and hippocampus (Min et al., 2020).

### 3.2 Bovine milk-derived EVs (BMEVs)

To effectively transfer LNA ASOs into the systemic circulation following oral delivery by oral gavage, bovine milk-derived EVs (BMEVs) were employed as a carrier on hPSC-derived neurons and primary human cells. Thus, leading to a slight elevation of cellular LNA ASO internalization and target gene reduction. LNA ASOs loaded BMEVs were found to be strongly biocompatible and non-toxic within the test concentration range. Their apparent loading efficiency is influenced by the type of nucleic acid utilized (e.g., siRNA vs. plasmid DNA vs. ASO) and the extent of purification and isolation. Thus, to overcome these challenges and to fully assess the potential of BMEVs for drug delivery purposes, additional critical and scientifically robust research along with relevant controls are required to overcome its challenges (Grossen et al., 2021).

### 3.3 Neurotensin-mediated targeted delivery

Neurotensin-mediated targeted delivery has the potential to improve the potency of ASOs with various nucleic acid alterations. Neurotensin (NT) receptors are found in the CNS. Due to its anionic nature, the neutrally charged morpholino ASO was interfering with the binding and internalization of neurotensin ASO. To overcome this issue, SAR studies were conducted and as a result of it, a peptide-based linker was recognized, which led to the development of an extended neurotensin peptide ASO conjugate. An ICV injection of neurotensin peptide conjugates was reported to increase cellular uptake and activity in SORT1 HEK293 cells and the spinal cord of mice. Additionally, it improved morpholino ASO potency to correct the splicing of survival motor neuron (SMN2) pre-mRNA. Although the reported potency gain was modest, the data provided in this research show that neurotensin peptide conjugation is a realistic method for enhancing the activity of ASOs in the CNS. Further optimization of the receptor-binding properties of the peptide ligands could result in greater potency enhancements for modulating gene expression using nucleic acid therapeutics in the CNS (Nikan et al., 2020).

### 3.4 Exosome-mediated delivery

Exosome-mediated ASO delivery (exo-ASO) a safe and highly effective method developed by yang et al., revealed lower toxicity and increased cellular uptake of ASO in primary neuronal cultures. Additionally, *in vitro* studies of exo-ASO resulted in significant attenuation of α-syn aggregation. While the *in vivo* studies revealed similar results along with a significant reduction in the expression of α-syn. Additionally, ASO ameliorated the degeneration of dopaminergic neurons in mice. Followed by improved locomotor function. Overall, the study reveals the immense potential of exosome-mediated ASO delivery as an effective therapeutic approach for PD (Yang et al., 2021).

## 4 *In vivo* studies of ASOs

To date, several ASOs-based preclinical and clinical studies are reported to explore the therapeutic potential of ASO (Table 2), amongst them an interesting study demonstrated the use of alpha-syn targeting 1227-ASO or 1233-ASO which were transfected into M17-EV and M17-syn cells revealing the superiority of 1233-ASO and was chosen ahead for *in vivo* studies. Further, 1233-ASO conjugate with indatraline hydrochloride (IND) (IND-1233-ASO) was prepared and administrated intranasally. It showed cellular selectivity, efficacy, and no cellular toxicity in DA neurons of animals and also has a high translational value in the treatment of PD with reduced alpha-syn expression (Alarcón-Arís et al., 2018). Similarly, another set of IND-ASO conjugates was prepared such as IND-1337-ASO [5′-CGCCTTCCACGGTTUUCU-3′], IND-1233-ASO [5′-CUCCCTCCACTGTCUUCU-3′], and IND-1227-ASO [5′-CCGTATCGTAAGCAGTAC-3′]. The ASO used here has four 2′-O-methyl RNA bases at both ends to protect the internal DNA from nucleus degradation and improve the binding to the target sequence. These ASO were administrated both intracerebroventricular (ICV) and intranasally to mice *via* a subcutaneously implanted micro-osmotic pump. Along with this non-human primate, treatments were also been performed. It was observed that both ICV and intranasal treatments with IND-1337-ASO prevented h-α-syn mRNA and protein accumulation in the SNc/VTA of the AAV5 mouse model, without affecting endogenous α-syn level. Importantly, the 30–40% decrease of h-α-syn mRNA levels obtained after ICV treatment (∼20% reduction after intranasal administration) did not induce DA neurotoxicity (Alarcón-Arís et al., 2020). Further extending the study, the ICV IND-1223-ASO treatment was found to prevent the accumulation of h-α-Syn mRNA and protein in the SNc/VTA and locus coeruleus (LC) of transgenic mice, without affecting endogenous m-α-Syn mRNA level. This study shows the benefit of optimizing conjugated ASO molecules as a disease-modifying therapy for PD, something that might be attractive in conjunction with current immunotherapy trials targeting α-Syn protein, or those with anti-aggregation agents (Pavia-Collado et al., 2021). Extending this study ahead research was been conducted to investigate IND-ASO (1,337 sequence) effects on the raphe α-synucleinopathy model. Primarily, mice were injected with AAV5 into their raphe nuclei and were subjected to treatment with vehicle or IND-ASO *via* ICV injection. Following the end of treatment, h-α-Syn mRNA expression in raphe 5-HT neurons was reduced by around 35% in mice treated with IND-ASO and AAV5 compared to mice treated with vehicle and AAV5. The IND-ASO therapy did not affect murine α-Syn mRNA expression or the amount of TPH-positive cells, indicating the safety and specificity of the ASO sequence used. Furthermore, IND-ASO-treated mice had lower quantities of h-α-Syn and p-α-Syn protein levels (31% and 65%, respectively) in the raphe nuclei as compared to vehicle-treated mice. As a result, this mouse model could be used to examine the early phases of PD pathogenesis, which are expected to involve derangements of the 5-HT system. This study also proved that IND-ASO administration selectively inhibited h-α-Syn production in 5-HT neurons, leading to less protein buildup in the forebrain and, as a result, a gain in 5-HT function and behavioral phenotype was observed. The current study used this to develop a technique in which the oligonucleotide was covalently linked to a monoamine transporter inhibitor (e.g. IND) for selective delivery to monoaminergic cells (Miquel-Rio et al., 2022).

**TABLE 2:** *In vivo* studies of ASOs.

ASO	Modification	Cells	Model	Route	Delivery	Ref.
IND-1233-ASO	Indatraline hydrochloride conjugation	M17-EV and M17-Syn cells	Mouse model	Intranasal	—	Alarcón-Arís et al. (2018)
IND- 1337-ASO [5′-CGCCTTCCACGGTTUUCU-3’]	Indatraline hydrochloride conjugation	Phospho-ser129-α -syn + cells	AAV5 Mouse model, monkeys	ICV and intranasal	Micro-osmotic pump	Alarcón-Arís et al. (2020)
IND-1233-ASO [5′- CUC​CCT​CCA​CTG​TCU​UCU-3’]						
IND-1227-ASO [5′-CCGTATCGTAAG-CAGTAC-3’]						
IND-1233-ASO	Indatraline hydrochloride conjugation	mouse α-Syn (m-α-Syn, 411-447 sequence)human α-Syn (h-α-Syn, 2,498-2,548 sequence)	Transgenic A30P*A53T*α-Syn mice	ICV	—	Pavia-Collado et al. (2021)
IND- 1337-ASO [5′-CGCCTTCCACGGTTUUCU-3’]	Indatraline hydrochloride conjugation	(TPH)-positive cells, non-TH-positive cells, phospho-S129-α-Syn (p-α-Syn) cells	AAV5 Mouse model	ICV	Osmotic mini pumps	Miquel-Rio et al. (2022)
ASO1 [5′-TTTAATTACTTCCACCA-3′]	Two 2′-O-methoxyethyl/DNA gapmer ASOs	SH-SY5Y human cells	Transgenic mice	ICV	—	Cole et al. (2021)
ASO2 [5′-CTGTTAAGTCACAAGCA-3′]						
hASO1 [5′-GTTTTCATCAATATCTGCAA-3′]						
hASO2 [5′-ACAGATATTTTTGTTCTGCC-3′]						
ASOA19 - AmNA-ASOs (3AmNA-9DNA- 2AmNA-1DNA)	AmNA modification	HEK293 cells	Transgenic animal PD models	ICV	—	Uehara et al. (2019)
	LNA modification					
ASOL19 - LNA-ASOs (3LNA-9DNA- 2LNA-1DNA)						
ASO1 [5′-GGA​CTG​CTC​TCT​TTC​TCA​CA- 3′]	Phosphorothioate oligonucleotides containing 2′-MOE groups at positions 1–5 and 15–20	SH-SY5Y cells	Wildtype C57BL/6J mice	ICVB injection, Subcutaneous bolus injection	—	Zhao et al. (2017)
ASO2 [5′-TCC​ACA​TTT​CTG​AAT​CCC​AG- 3′]						
FAM-ASO4 -GCTCCCTCCACTGTCT	2 locked nucleic acids on the 5′ and 3′ termini containing 2′-O-methoxyethyl modifications and phosphorothioate backbone	SH-SY5Y cells HEK293- α-syn cells	α-syn A53T mouse	ICV	Exosomes	(J. Yang et al., 2021)
Antisense caveolin-1 phosphorothioates [5′-TTTACCCCCAGACAT-3′]	Phosphorothioate oligonucleotide	B103 cells (mouse neuroblastoma lines)	—	—	—	Hashimoto et al. (2003)
ASO [5′-TTT​AAT​TAC​TTC​CAC​CA- 3′]	—	—	C57BL/6J (WT) male mice	ICV infusion	—	Boutros et al., (2021)
ASO [5′-CCTTTCATGAACAC ATCCATGGC-3′]	Phosphorothioate group at every residue	Hippocampal cultures	Sprague Dawley rat Embryos	—	—	Rueter et al. (2000)

A study involved the use of two 2′-O-methoxyethyl/DNA gapmer ASOs targeting Snca for *in vitro* and *in vivo* experimentation in addition to a control ASO. Sequences used were ASO1 [5′-TTTAATTACTTCCACCA-3′], ASO2 [5′-CTGTTAAGTCACAAGCA-3′] and CTL ASO [5′-CCTATAGGACTATCCAGGAA-3′]. After 70 days of single ICV administration of ASO1 was found to reduce SNCA mRNA by approximately 50%, prolonged the duration of action, and prevent pathogenic α-Syn aggregate deposition in an *in vivo* PFF model of PD. SNCA ASO2 also reduced pSer129 + aggregate counts but to a lesser extent than ASO1. The study demonstrates that ASO-mediated suppression of SNCA prevented and reversed the progression of α-Syn-mediated pathology in rodent transmission models of PD, demonstrating the potential of SNCA ASOs as a therapy for PD patients. Long-term studies with sustained reduction of α-Syn prevented and even delayed pathology and associated TH loss. Furthermore, central delivery of human SNCA ASOs (hASO1 [5′-GTTTTCATCAATATCTGCAA-3′], hASO2 [5′-ACAGATATTTTTGTTCTGCC-3′]) reduced expression of mRNA and protein throughout the brains of both the humanized mouse and non-human primates (NHPs), demonstrating that human SNCA ASOs are active in regions of the brain susceptible to PD in a larger species (Cole and Daley 2021). However, yet another study demonstrated the use of gapmer (13–15-mer phosphorothioate oligonucleotides) which was modified to design AmNA-ASOs (15-mer chimeric antisense oligonucleotides) containing five AmNA modifications. Later, AmNA-ASOs were transfected into human embryonic kidney 293 (HEK293) cells and after 24 h quantification of SNCA mRNA level was carried out using quantitative polymerase chain reaction (qPCR) in a reduction in SNCA mRNA levels was significantly observed particularly with AmNA-ASO No.19 (ASOA19). Further extending the study LNA-ASO No. 19 (ASOL19) was generated having the same target sequence as that of ASOA19 and both ASOA19 and ASOL19 were compared for their knockdown efficiencies *via* transfecting them into HEK293 cells. The findings indicate that AmNA-modified ASO knocked down SNCA mRNA more effectively than LNA-modified ASO. Thus, it was observed that AmNA-ASO can ameliorate several motor dysfunctions seen in PD model mice by lowering SNCA mRNA and the related protein levels when administered intracerebroventricularly. Research data shows that AmNA-ASO has the potential to be a cutting-edge treatment for PD and other synucleinopathies with no off-target effects (Uehara et al., 2019). In a study, Lrrk2 mRNA was measured using reverse transcription-quantitative polymerase chain reaction (RT-qPCR), and ASOs complementary to Lrrk2 mRNA were designed and screened in SH-SY5Y cells. LRRK2-targeted sequences (SEQ1, SEQ2) were homologous to the mouse and human LRRK2 genes. It was further tailored for use in primary neurons and *in vivo* and was subsequently given the names ASO1 and ASO2. SEQ1 and SEQ2 respectively were 20-mer gapmer phosphorothioate oligonucleotides with 2′-MOE groups at positions 1–5 and 15–20. ASO1 and ASO2 had identical sequences and chemistry designs to SEQ1 and SEQ2, except for their mixed phosphorothioate and phosphodiester backbones. For *in vivo* screening and characterizations of ASOs PBS, CTL ASO, or ASO1 or ASO2 were administered to wildtype C57BL/6J female mice as a single intracerebroventricular bolus (ICVB). However, for systemic studies, a subcutaneous bolus injection was administered. The acquired results showed that ASO-mediated suppression of endogenous LRRK2 decreased motor behavioral defects, decreased pathological aggregation of α-syn, and inhibited TH cell loss in the preformed α-syn fibrils (PFF) model (Zhao et al., 2017). Further, a study involved designing of FAM label ASO gapmers sequence containing locked nucleic acids (LNC) at each end flanking the central base of DNA along with 2′-O-methoxyethyl modifications, 15 unmodified central oligodeoxynucleotides to support RNase H activity, and a phosphorothioate backbone to improve nuclease resistance. The designed sequences were ASO1, ACTGCTGTCACACCCG; ASO2, GCTGTCACACCCGTCA; ASO3, CAACTCCCTCCTTGGC; ASO4, GCTCCCTCCACTGTCT; scrambled ASO, ACTCCCGAACCTGTCT, which were later transfected into SH-SY5Y cells. A thorough evaluation of phosphorylation and aggregation of α-syn caused by α-syn-preformed fibrils (PFFs) aggregation in HEK293- α-syn cells were conducted to ascertain if the decreased expression of α-syn by ASO4 could ameliorate α-syn aggregation. The outcomes showed that ASO4 decreases pathological phosphorylation and aggregate formation of α-syn. Thus, ASO4 was taken ahead of and was loaded in exosomes extracted from human bone marrow mesenchymal stem cells (MSCs) and injected into α-syn A53T mice *via* ICV delivery. The results obtained demonstrated that exo-ASO4 significantly reduced α-syn-related pathology in the PD mouse model, reduced dopaminergic neuron degeneration in the nigrostriatal system, and significantly eased motor dysfunction. Overall, the research indicates that exosomes exhibited low toxicity and had a strongly effective *in vivo* and *in vitro* distribution. Exosomes are therefore more suitable drug delivery vectors for ASOs than currently used commercial reagents (Yang et al., 2021). A study focused on a possible key player in the extracellular signal-regulated kinase (ERK) regulation of synaptic plasticity which is the caveolin-1-ERK pathway. Dysregulation of this pathway might be a critical step leading to neurodegeneration. To address this and to come up with a solution, α-synuclein-overexpressing cells were treated with caveolin-1 antisense phosphorothioates (p)-oligonucleotide (5′-TTT​ACC​CCC​AGA​CAT-3′; complementary to the 15-base initiation sequence of rat caveolin-1) and it showed to re-established ERK activity by up-regulating caveolin-1 expression in B103 neuroblastoma cells. This finding is crucial because the caveolar signaling system may be a common therapeutic target for many debilitating diseases since caveolar dysfunction might play a ubiquitous role in the pathogenesis of various neurodegenerative disorders, including PD (Hashimoto et al., 2003). According to a study, LB-like pathology was induced in C57BL/6J (WT) male mice *via* intracranial injection. After 3 months from the time of injection, animals were subjected to cognitive and behavioral testing. Later at 4 months from the time of injection, mice were subjected to ICV infusion of either a mouse sequence α-syn-targeted ASO or a scrambled oligonucleotide which served as control. After, 5 weeks again cognitive and behavioral testing was performed to assess the effects of the targeted ASO. The results indicated that preformed fibrils (PFF) injections in the motor cortex are linked to mild, progressive deficits and that this particular ASO can successfully decrease alpha- and phosphorylated synuclein protein levels. LB-like pathology in mice was found to be reduced months after a single injection, however, there were also detrimental, off-target side effects observed (Boutros et al., 2021). A study involved the treatment of hippocampal neuronal cultures with oligonucleotides containing a phosphorothioate group at every residue. Oligonucleotides were prepared from the 5′ end of the coding region for sprague dawley rat α-synuclein. The sequences used were ASOs (5′-CCT​TTC​ATG​AAC​ACA​TCC​ATG​GC-3′), reverse sense oligonucleotide (5′-GCC​ATG​GAT​GTG​TTC​ATG​AAA​GG-3′), and a scrambled control oligonucleotide sequence (5′- TAG​CTC​GCT​ACG​TAA​TCA​CCA​CT-3′). Based on the results obtained it was observed that ASO was able to reduce α-syn levels by ∼50% (Rueter et al., 2000).

Phase 1 single and multiple ascending dose studies (NCT03976349) were conducted with the primary objective to evaluate the pharmacokinetics, safety, and tolerability of LRRK2 ASO BIIB094, delivered intrathecally to adult PD patients is ongoing. It is aimed at PD patients with or without LRRK2 mutations and was started by Biogen in partnership with Ionis. The second objective of this study was to assess the pharmacokinetic profile of the ASO. In the near future, Biogen, a collaborator with IONIS on multiple BIIB projects, will begin phase 1 research for their lead ASO BIIB101 (NCT04165486 and EudraCT Number 2019–001105-24), however, the target is not yet been revealed. Considering the primary objective the target is likely to be SNCA mRNA (Doxakis 2020).

## 5 Safety assessment of ASOs

The most common safety concern is raised regarding adverse side effects of ASOs based treatment approaches. The first consideration is “on-target” toxicity due to lower levels of a total protein and it is associated with a wide range of potential problems which remain unknown and contribute to a major challenge to predict in the adult human. Thus, in preclinical and clinical testing, ASOs will continue to be assessed for their on-target toxicity. The second most crucial concern is “off-target” toxicity as the body recognizes it as foreign DNA and leads to activation of the immune system (Miller and Schoch 2017).

Furthermore, *in vitro* screening for these effects can be used to eliminate ASOs that cause an immunological response (Kuijper et al., 2021). Extensive research and development have mitigated some of the worries about adverse side effects. However, there are still on-target and off-target concerns to address, some of which may not be recognized yet (Miller and Schoch 2017). Thus, safety considerations can be classified into two types first is sequence-specific safety for example exaggerated pharmacology can be a problem in ASO-based treatments aimed at protein knockdown, where too much knockdown of the target transcripts and encoded proteins in the target tissue, or non-target tissue, can lead to undesired effects, and safety is a big worry. The chemical modifications of the ASOs are the second safety concern due to their different safety profiles which also must be taken into consideration (Kuijper et al., 2021). Another safety issue associated with ASOs is drug-induced thrombocytopenia; it has been observed in preclinical and clinical trials after numerous ASO administrations. There have been two types of thrombocytopenia discovered. Mild, transient, and dose-dependent thrombocytopenia is the more common type, which has been observed in 10% of 2′-MOE PS ASOs (Chi et al., 2017). The Oligonucleotide Safety Working Group (OSWG) has issued comprehensive guidelines for evaluating ON safety. As more preclinical and clinical data become available, our understanding of ON-mediated toxicity improves. While the idea of class toxicity has become more sophisticated as an understanding of diverse chemistries has grown, ON-related side effects still fall into two categories: 1) hybridization-dependent effects, which include on- and off-target effects, and 2) hybridization-independent effects, which are mostly mediated by protein-binding properties (Hammond et al., 2021).

## 6 Role of miRNAs and their biogenesis

In 1993, *Caenorhabditis elegans* revealed the presence of the first two miRNAs, let-7 and lin-4. To date, about 2000 miRNAs have been discovered and reported on http://www.mirbase.org (accessed on 22 February 2022). It is believed that miRNAs play crucial roles in numerous biological processes under both pathological and physiological conditions (Nguyen et al., 2022). miRNAs can be employed in the diagnosis and treatment of various diseases because of their high tissue and cell specificity. miRNAs have the power to modulate more than half of all coding genes, making them central to the biological process stability. As a result, it is speculated that miRNAs may have a role in neurodegenerative disorders (Herranz and Cohen 2010; Krol et al., 2010; Gascon and Gao 2012; Liu et al., 2022). Certain miRNAs are dysregulated in the brains of patients with neurodegenerative disorders, such as PD, suggesting that miRNAs may play a critical role in the etiology of these disorders. miRNAs play a very important role in the regulation of expression of their mRNA targets either by inhibition of translation or by degradation of mRNA. A study demonstrated that in the ventral midbrain of old mice, dicer expression is diminished. Additionally, mice with targeted deletion of the dicer gene in dopaminergic neurons allegedly experienced a growing loss of dopaminergic (DA) neurons along with serious locomotor impairments. The latter finding suggests that Dicer is essential for DA neuron survival and that loss of mature miRNAs due to deletion of the Dicer gene may be involved in the development and/or progression of PD (Nakamori et al., 2019). MicroRNAs (miRNAs) belong to the class of endogenous short non-coding RNAs with a length of 20–25 base pairs and no open reading frame (ORF). Knocking down requires the complex formation of miRNA guide strands with silencing proteins to bind to the complementary sequences in the target mRNA’s 3′-untranslated region (3′-UTR). This silencing complex will eventually result in translational repression or mRNA breakdown (Ha and Narry Kim 2014). The pathways associated with MiRNA biogenesis are categorized as: canonical and non-canonical.

**FIGURE 4:**
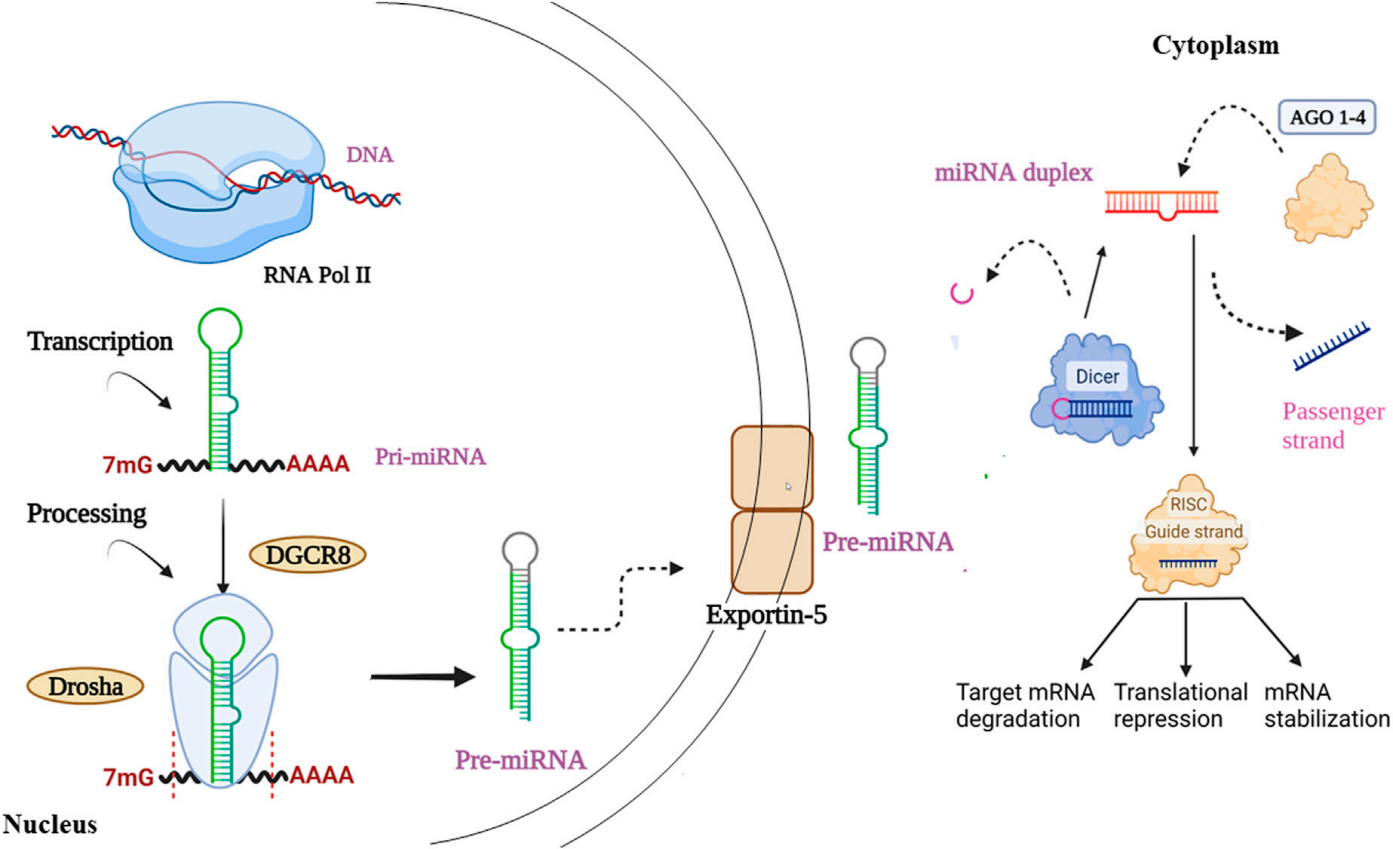
Starts right from the nucleus involving sequential production of **(a)** pri-miRNA **(b)** pre-miRNA, followed by transportation of pre-miRNA into the cytoplasm. Furthermore, involving the genesis of miRNA duplex, its binding with AGO protein and finally resulting in the generation of mature miRNA.

Canonical biogenesis is the dominant pathway for miRNA processing. It begins right from the nucleus (Figure 4). The RNA polymerase II plays a vital role in transcribing miRNA genes, producing an imperfect stem-loop structure with hundreds to thousands of nucleotides flanked by single-strand (ss)RNA that is capped on the 5′ end and polyadenylated on the 3′ end - pri-miRNA (Cai et al., 2004; Lee et al., 2004). Inside the nucleus, the pri-miRNAs are processed, where the DiGeorge Syndrome Critical Region 8 (DGCR8), a ribonuclease III enzyme Drosha, and associated proteins build a microprocessor complex that produces precursor miRNAs (pre-miRNAs) (Denli et al., 2004). Owing to their hairpin structure, pre-miRNAs are labelled ‘mir’ and are more stable than pri-miRNA. Exportin-5 identifies 2-nucleotide projected from the hairpin loop of pre-miRNAs and is then transferred to the cytoplasm. It is then cleaved by the RNase III enzyme Dicer processes them to form miRNA duplexes by choosing one strand due to its greater thermodynamic stability. The argonaute proteins of the RNA-induced silencing complex (RISC) then recognize the miRNA duplex. Further, RISC generally binds to one of the strands with lower 5′ stability or 5′ uracil, called the guide strand, forming the mature miRNA designated as “miR”. The mature guide strand comprises 20–22 nucleotides and forms a functional RNA-induced silencing complex (RISC) with argonaute proteins AGO 1–4 (Ha and Narry Kim 2014; Kumar and Reddy 2016; Macgregor-Das and Das 2018; Michlewski and Cáceres 2019; Paul et al., 2020; Sun et al., 2018). During the biogenesis process, the passenger strand is the unloaded strand that will be unwound from the guide strand through various mechanisms depending on the degree of complementarity. miRNA passenger strands with no mismatches are cleaved by AGO2 and degraded by cellular machinery. On other hand, miRNA duplexes with central mismatches or non-AGO2 loaded miRNA are unraveled and degraded passively (Ha and Kim 2014). The mature miRNA–RISC complex represses gene expression by complementary base pairing with the target mRNA’s 3′ UTR region. The repression of target gene expression is largely dependent on the extent of complementarity of the seed region (nucleotide 2–8) to the target mRNA. Perfect complementarity causes mRNA destruction, while partial complementarity causes translational repression (Table 1) (Baek et al., 2008; Zeng et al, 2003). Furthermore, noncanonical miRNA biogenesis pathways can also be used to generate functional miRNAs. Such as mirtrons are produced by pre-mRNA splicing, and miRNAs are produced by short nucleolar RNA (snoRNA) precursors (Ha and Kim 2014).

For the identification of miRNAs and their role, a correct naming scheme is very crucial. The miRNAs produced through the biogenesis process may have a borderline similarity. In such situations, the naming scheme is a bit tricky where two distinct mature miRNA sequences seem to have emerged from the same hairpin precursor’s opposing arms, such that the two different miRNAs can be named as miR-17-5p (5′ arm) and miR-17-3p (3′ arm) depending upon their respective arm of resection. Generally, a prefix of 3 or 4 letters is used to denote species, such that the prefix ‘hsa’ is used for species “*Homo sapiens*” and the miRNA will be identified as hsa-miR-101 (Griffiths-Jones et al., 2006).

## 7 miRNA-based therapeutics

The discovery of miRNAs as critical regulators of gene expression in a variety of disorders, including PD, has made miRNAs based targeting a viable therapeutic option. Scientific interest in miRNAs has always been centered around two areas of investigation: 1) miRNAs as potential disease biomarkers, and 2) miRNAs as a molecular target involved in various disease therapeutics. MiRNAs have been thoroughly explored as a promising therapeutic medication class which demonstrated outstanding results in cancers and many other disorders, including neurodegenerative diseases since the first miRNA replacement therapy, began phase 1 clinical trials in ClinicalTrials.gov (2013b) (Wen 2016; Angelucci et al., 2019). According to estimates, 70% of known miRNAs express themselves in the brain and regulate crucial signaling pathways important for synaptogenesis, neurite outgrowth, neural plasticity, and memory functions (Nadim et al., 2017). Since miRNAs possess various advantages such as one single miRNA can regulate multiple genes and can be utilized to silence up-regulated genes in PD by connecting the 3′ untranslated regions (3′UTR) of a gene. Further, its size of ∼22 nucleotides is beneficial for the design of miRNA drugs. The post-transcriptional regulation of miRNAs appears to play an essential role in the regulation of gene expression, according to emerging evidence (Li et al., 2017; Majdi et al., 2016). The miRNA-based therapeutics can be classified as miRNA inhibitors and replacement therapy. Aberrant expression of miRNA may lead to disease so miRNA inhibitors can be used to downregulate the expression of dysfunctional miRNA. Replacement therapy can be used to re-establish the lost miRNA levels within the diseased patients (Bernardo et al., 2015). To restore the suppressed miRNA level miRNA mimics (agonist) can be used, and on other hand to repress overactive miRNA function anti-miR (antagonist) can be used to inhibit miRNA function (Rooij and Kauppinen 2014). The major hurdle faced by these therapeutics is the instability issues faced due to enzymatic degradation, and facilitating entry by crossing the blood-brain barrier. Thus, a safe and stable delivery system would be required for miRNA-based therapeutics. Moreover, multiple drug delivery systems can be used for the delivery of miRNA-based therapeutics that have already been authorized for human use (Sun et al., 2018; Wen 2016). Thus, this could be used as a potential therapeutic approach for PD. The current findings imply that α-synuclein build-up induces microRNA-mediated aberrant cell cycle activation in post-mitotic dopaminergic neurons. As a result, the mitotic cell cycle pathway at the level of miRNAs may present promising new treatment targets for PD (Findeiss et al., 2021).

### 7.1 miRNA inhibitors (Anti-miRNA/AntagomiRs)

The miRNA inhibitors are known as antagomirs or anti-miRNAs, these may be genetically or chemically prepared (Mattes et al., 2007; Yang and Mattes 2008). Artificial miRNA inhibitors are partially or completely complementary oligonucleotides that bind to miRNAs and block their function, allowing various genes to be expressed. miRNA inhibitors (antagomirs) inhibit miRNA from interacting with the miRISC or the miRISC from interacting with its target mRNAs. These antagomirs either block mRNA from being translated into protein or induce its degradation (Zhang, 2013). It was reported that an i. c.v. injection of AntimiR-134 + Neuromag® may exhibit neuroprotective activity in the rat striatum, a region critically involved in PD neuropathology (Table 3). It was observed that miR-134 knockdown was triggered by antimiR-134 a sequence-dependent and target specific, which is crucial for achieving a further miRNA-based neuroprotective therapy (de Almeida et al., 2018). A study revealed that miR-30c-5p is upregulated in PD patients (Vallelunga et al., 2019). Further, another study also showed that miR-30c-5p overexpression enhances neuronal damage by lowering autophagy levels and negatively regulating ATG5 expression in PD mice and cell models, while miR-30c-5p antagomir could reduce the damage to dopaminergic neurons by upregulating ATG5 expression. Thus, miR-30c-5p can be a potential target for PD treatment (Zhang et al., 2021). Yet another potential target for the treatment of PD is mir-3473b. According to a study, injecting mir-3473b antagomir increases the *in vivo* expression of TREM2 and ULK1 in MPTP-treated mouse SNpc and regulates the impact of autophagy in the pathogenesis of PD. Further, the data suggested that inhibition of mir-3473b can inhibit neuroinflammation during the development of PD disease (Lv et al., 2021). Thus, various miRNA inhibitors can become the future of miRNA-based therapeutics for PD. According to a study it was observed that injecting antagomiR-421 into SNpc-protected DA neurons in 6-OHDA-treated PD mice, negatively regulates the expression of myocyte enhancer factor 2D (MEF2D) by directly binding to MEF2D mRNA. Antagomir-421 inhibits miR-421 which was upregulated in PD pathogenesis and also preserved the Bcl2/Bax system which was disrupted in the 6-OHDA-treated PD mice model. The Bcl2/Bax ratio is a common indicator used to assess apoptosis. Thus, preserving the Bcl2/Bax system and re-establishment of MEF2D-protected neurons from neurotoxicity in both animal and cellular PD models makes it a novel approach and also provides potential targets for PD theragnostic (Dong et al., 2021).

**TABLE 3:** Pre-clinical and *in vitro* data of miRNA-based therapeutics for PD.

miRNA	Model	Type of dysregulation	Treatment approach	References
miR-134	Rat striatum	Upregulated	AntimiR-134 + Neuromag®	de Almeida et al. (2018)
miR-30c-5p	PD mice and cell model	Upregulated	miR-30c-5p antagomir	(Zhang et al., 2021)
miR-3473b	MPTP-treated mouse SNpc	Upregulated	mir-3473b antagomir	Lv et al. (2021)
miR-421	6-OHDA-treated PD mice	Upregulated	AntagomiR-421	Dong et al. (2021)
miR-7	mouse striatum	Downregulation	miR-7 mimics	(Zhou et al., 2016)
miR-30e	MPTP-treated mice	Downregulation	miR-30e agomir	Dongsheng Li et al. (2018)
miR-135b	MPP + PD modelled SH-SY5Y and PC-12 PD cells	Downregulation	miR-135b mimics	(Zeng et al., 2019)
miR-425	MPTP-treated mice	Downregulation	AgomiR-425	(Hu et al., 2019)
miR-543-3p	PD mice	Downregulation	Lentivirus-containing antisense miR-543-3p	Wu et al. (2019)
miR-150	LPS-treated BV2 cells	Downregulation	miR-150 mimics	(Li et al., 2020a)
miR-29c-3p	*in vitro* PD mouse	Downregulation	miR-29c-3p mimics	(Wang et al., 2020)

Anti-miRNAs are chemically modified as 2′-O-Me (methylation) and 3′-cholesterol-conjugated oligonucleotides to become antisense oligonucleotides exhibiting full complementarity to the mature miRNA. Their specificity is defined by high binding affinity and given their therapeutic potential, these are suitable for use in clinical studies. This novel technique silences endogenous miRNAs in an efficient, specific, and long-lasting manner (Davis et al., 2006; Lima et al., 2018).

### 7.2 miRNA mimetics (AgomiR)

The short double-stranded oligonucleotides known as miRNA mimics function as miRNA precursors. These are detected by miRNA biogenesis machinery as soon as they are delivered into cells, and are subsequently processed suitably (Latronico and Condorelli 2011). The miRNA mimetics also known as agomiR, are artificial miRNAs duplex that function as an endogenous counterpart; thus, they do not need to be processed and they directly binding to the RISC complex to interact with their respective mRNA targets *in vivo* and provide a mimetic effect on the phenotype of an organism (Bernardo et al., 2015; Goldgraben et al., 2016; Lin et al., 2018). A study demonstrated that stereotactic injection of miR-7 mimics into the mouse striatum has been found to diminish neuroinflammation and provide neuroprotective benefits in MPTP-lesioned mice by targeting the 3′-UTR of NOD-like receptor protein 3 (NLRP3) mRNA (Table 3) (Zhou et al., 2016). It was reported that miR-30e agomir has the ability to increase miR-30e expression in MPTP-treated mice and it remarkably improves motor behavioral deficiencies and neuronal activity and inhibits the loss of dopamine neurons. Further, it also alleviates the enhanced α-synuclein protein expression in SNpc of MPTP-PD mice. Additionally, MiR-30e reduces NLRP3 inflammasome activity, which reduces neuroinflammation in the MPTP PD model. Thus, miR30e may be a key inflammation-mediated molecule that could be a potential target for PD therapeutics (Li et al., 2018). Like these, there are several other agomir that can act as a prospective therapeutic approach for PD patients. Such as miR-135b mimics showed a reduction in the detrimental consequences of MPP + PD *in vitro* model (SH-SY5Y and PC-12 PD cells) on pyroptosis, by downregulating the NLR family pyrin domain containing 3 (NLRP3) and Caspase-1 genes (Zeng et al., 2019). A study revealed that miR-425 deficiency in PD induces necroptosis of dopaminergic neurons, and targeting miR-425 *via* intracerebral administration of agomiR-425 in MPTP-treated mice re-establish dysfunctional dopaminergic neurodegeneration and restores locomotor impairments. Thus, agomiR-425 can be a probable therapeutic approach for neuron loss (Hu et al., 2019). A study reported that lentivirus-loaded antisense miR-543-3p was administered locally and unilaterally into the substantia nigra (SN) of PD mice leading to improved motor function and lowered DA neuronal damage and α-syn aggregation levels (Wu et al., 2019). It is reported that the serums of PD patients have downregulated miR-150 levels which negatively corresponds to proinflammatory cytokine levels (IL-6, IL-1β, and TNF-α) and administration of miR-150 mimics restores the miR-150 levels in lipopolysaccharide (LPS)-treated BV2 cells. This leads to a reduction in above mentioned proinflammatory cytokines by targeting the AKT3 gene (Li et al., 2020). *In vitro* PD mouse models, miR-29c-3p mimics prohibited microglia activation and restrained the NLRP3 inflammasome by directly targeting the nuclear factor of activated T cells 5 (NFAT5) (Wang et al., 2020). These were some of the miRNAs which can act as a potential target for miRNA-based theragnostic.

## 8 Impact of miRNA on α-synuclein

miRNAs may play a crucial role as a therapeutic approach for PD. The survival of neurons involved in the pathophysiology of PD depends on the quantitative detection of specific pathogenic proteins (Maciotta, 2013). A study demonstrated that six miRNAs (miR-7, miR-153, miR-34b, miR-34c, miR-214, and miR-1643) directly bind to the 3′- untranslated region (UTR) of the α-synuclein mRNA transcript and negatively regulate its expression. miRNAs both regulate and are regulated by α-synuclein expression, indicating the complex network between miRNAs and α-synuclein (Recasens, 2016). Among them, miR-7, miR-34b, and miR-34c were shown to be reduced in PD brains, implying that reduced expression of these specific miRNAs in PD brains can lead to raised α-synuclein levels and assist in PD pathogenesis. Through its 3′-UTR, miRNA-153 downregulates α-synuclein expression (Junn et al., 2009; Doxakis 2010). It suppresses the expression of α-synuclein at both the mRNA and protein levels. As seen in the 1-methyl-4-phenylpyridinium (MPP+) *in vitro* model of PD, increased expression of miRNA-153 exhibits neuroprotective effects over dopaminergic cell types (Doxakis 2010; Fragkouli and Doxakis 2014; Titze-de-Almeida and Titze-de-Almeida 2018). A total of four studies looked into the role of miR-153 in α-synuclein expression and its protective effects (Doxakis 2010; Fragkouli and Doxakis 2014; Lim and Song 2014). Two studies looked at the combined effects of both miR-7, miR-153, and the miR-7/153 combination (Doxakis 2010; Fragkouli and Doxakis 2014). According to one study, miR-1643 directly regulates the expression of α-synuclein (Lim and Song 2014). A luciferase assay in 293 TF cells was used to validate the direct binding of miR-1643 to the α-synuclein 3′-UTR region (Zhang et al., 2014). Initially, a study identified miRNA-7 to bind to the 3′-UTRs of α-synuclein mRNA and downregulate its expression. The findings imply that miRNA-7 regulates the Brain-derived neurotrophic factor (BDNF)/α-syn axis in the early stages of PD and has the potential to be employed as a biomarker or therapeutic target. MiRNA-7, which is mostly expressed in neurons, represses α-synuclein expression and protects cells from oxidative stress (Doxakis 2010). Increased expression of miRNA-7 has been demonstrated to have neuroprotective effects on dopaminergic cell types in the 1-methyl-4-phenylpyridinium (MPP+) *in vitro* model of PD (Doxakis 2010; Fragkouli and Doxakis 2014; Titze-de-Almeida and Titze-de-Almeida 2018). In one study, the miR-7T-AsRed target vector was used in the model, which was able to promote the accumulation and aggregation of α-synuclein. As a result, α-synuclein expression and oligomerization increased, as did dopaminergic cell death and a decrease in striatal DA (McMillan et al., 2017). MiRNA-7 is distinct among α-synuclein targeting miRNAs in various ways. In addition to targeting the 3′-UTR of α-synuclein mRNA, miRNA-7 promotes autophagy, which aids in the clearance of preformed α-synuclein aggregates. Notably, in addition to modulating α-synuclein levels and processing, miR-7 also has a function in neuroinflammation (Zhang and Cheng 2014). According to one study, miR-34b and miR-34c directly target α-synuclein expression. The overexpression of miR-34b or miR-34c in SH-SY5Y cells, and direct binding interaction between miR-34/miR-34c and α-synuclein resulted in a considerable reduction in α-synuclein mRNA and protein levels. Among the miRNAs linked with both the substantia nigra and the putamen, it was discovered that hsa-miR-34b and hsa-miR-95-hsa-miR-34b are connected with a decrease in α-synuclein expression (Kabaria et al., 2015). Identifying miRNAs that are differently expressed in post-mortem PD brain tissue and nonaffected controls aids in understanding the role of miRNAs in the etiology of PD and may lead to the identification of prospective treatment options (Nakamori et al., 2019). Certain miRNAs have been found to be considerably increased in the brain tissues of PD patients. Amongst those miRNAs, a few of them such as miR-21*, miR-26b, miR-224, miR-373*, miR-301b, and miR-106b can target the chaperone-mediated autophagy pathway. Any kind of impairments to this pathway may interfere with the breakdown of the α-synuclein protein and contribute to the pathogenesis of Lewy bodies (Alvarez-Erviti et al., 2013).

Previously, the profiling of miRNA performed on the midbrains of PD and control subjects demonstrated that miR-133b is highly enriched in the midbrain but found to be low in PD brains. However, another study later challenged this conclusion by demonstrating that miR-133b levels in midbrain tissue from PD brains remained unaltered (Doxakis 2010). Ablation of miR-155 in mice are protected from DA neurons degenerating due to α-synuclein, which results in less microgliosis (Thome et al., 2016). In an α-synuclein-driven PD model, miR-155 deletion prolonged lifespan in SOD1G93A mice and prevented experimental autoimmune encephalomyelitis (EAE) induction, as well as decreased microgliosis and neuronal loss (Thome et al., 2016; Connell et al., 2011; Murugaiyan et al., 2015; Hu et al., 2013; Ksiazek-Winiarek et al., 2017; Mycko et al., 2015; Zhang et al., 2014; Zhang and Braun 2014; Zhang and Braun 2015). LAMP2A could enhance chaperone-mediated autophagy, a key pathway for α-synuclein breakdown. By directly targeting the 3′-UTR of LAMP2A (lysosome-associated membrane protein 2), miR-21 upregulated the expression of α-synuclein, whereas a miR-21 inhibitor effectively downregulated α-synuclein and demonstrated neuroprotective effects (Xilouri et al., 2013; Su et al., 2016). MiR-21-5p downregulation appeared to have a neuroprotective effect, which was mediated by enhanced expression of the miR-21-5p target LAMP2A. LAMP2A increased α-synuclein autophagy, which limited pathogenic aggregation (Su et al., 2016). A study revealed that under both baseline and MPP + conditions, the overexpression of miR-133b downregulated the α-synuclein mRNA levels in PC2 cells and primary neurons *via* inhibition of RhoA by miR-133. Although this mechanism has not been experimentally validated. Further, aiding this information RhoA has previously been noted to directly inflect/regulates α-synuclein expression by activating the serum response element (SRF) transcription factor and the GATA-2 transcription factor, which modulates α-synuclein expression *via* occupancy at the intron-1 (Scherzer et al., 2008; Zhou et al., 2011). It was reported that in human dopaminergic neuroblastoma, SH-SY5Y cells treated with MMP+, the expression of miR-203a-3p, was down-regulated leading to reduced cell proliferation and triggering apoptosis in the SH-SY5Y cells then enhances the expression of SNCA, p53 and cleaved Caspase-3 proteins of which were inhibited by the up-regulation of miR-203a-3p. Apart from these other miRNAs were also identified as regulators of SNCA expression which include the miR-30b, miR-34b/c, miR-214, and miR-433 (Consales et al., 2018; Shen et al., 2020; Tarale et al., 2018; Wang et al., 2015; Wang et al., 2008) for the very first time demonstrated that MiR-433 has shown to directly target the FGF20 mRNA transcript and negatively regulates FGF20 protein translation. It was observed that when SH-SY5Y cells were treated with the miR-433 target FGF20, levels of α-synuclein protein were considerably higher than in control cells. According to the authors, FGF20, like FGF2, might regulate α-synuclein expression *via* FGF-receptor 1 (FGFR1) (Ohmachi et al., 2003; Rideout et al., 2003). Supporting this hypothesis, miR-433 did not bind to the α-synuclein 3′-UTR as reported with luciferase assays in Neuro2A and SK-N-SH cells (Schmitt et al., 2012). A study has shown that miR-214 has a direct effect on α-synuclein expression. MiR-214 has been found to directly target the α-synuclein 3′-UTR in SH-SY5Y cells using luciferase assays. Furthermore, miR-214 overexpression decreased α-synuclein expression at both the mRNA and protein levels, whereas miR-214 downregulation increased both α-synuclein expression (mRNA and protein) as well as the quantity of α-synuclein aggregates in cells (Wang et al., 2015). Apart from these sets of mRNAs, miRNA-16–1 in a human neuroblastoma cell line was found to promote aberrant α-synuclein accumulation in PD by targeting heat shock protein 70 (Zhang et al., 2014).

## 9 Challenges for nucleic acid-based therapeutics

Nucleic acid-based therapeutics including both ASO and miRNAs in spite of being a potential therapeutic strategy still it possesses several challenges which are needed to be addressed. The two major constraints such as targeted delivery and the choice of the delivery system to cross the BBB effectively or escape the renal excretion and ultimately reach the biological target while avoiding off-target side effects. For these, the ASO drugs must travel into the bloodstream, cross biological barriers, and be internalized by cells after administration. Subsequent to internalization and entrapment of ASOs in secretory vesicles must avoid lysosomal degradation (Juliano 2016; Linnane et al., 2019). However, the delivery vehicle for miRNA-based therapeutics must also be able to protect miRNA from degradation and impart stability and long shelf life to the miRNAs (Goh et al., 2019). Additionally, to improve ASO stability and targeting, many solutions have been devised and are still being developed (Thomas and Weber 2019). Such as chemical modifications, bioconjugation to various moieties, and the utilization of delivery vehicles (Gagliardi and Ashizawa, 2021). Some approaches such as conjugating liposomes with antibodies for delivering plasmid vectors also have enhanced brain penetration. To achieve site-specific drug delivery aptamers and monoclonal antibodies can be used in association with RNAi duplexes (Boado 2007; Juliano 2016). Thus, aptamers not only portray their role as theragnostic agents in NDD but it is also being used as the delivery agent for miRNA (Chandola and Neerathilingam 2020). A study involved the use of nanoparticles as a delivery system, demonstrated that intraventricular administration of miR-124 loaded nanoparticles *in vivo* increases the number of subventricular zone-derived neuroblasts in the striatum of 6-OHDA-treated mice and induces neuro-regeneration leading to brain repair (Saraiva et al., 2016). The miRNA delivery system employs both non-viral and viral vectors (Pereira et al., 2013). Adenovirus, retrovirus, and lentivirus are the most commonly used viral vectors (Yang 2015). The non-viral systems use liposomes and lipid nanoparticles (LNP)-mediated carrier systems (Lee et al., 2019). A PEG moiety conforms LNPs, an ionizable cationic lipid, i.e., 1,2-dioleyl-3-dimethylammonium propane (DODAP), resulting in better encapsulation efficiency than liposomes (Campani et al., 2016). Both viral and non-viral miRNA delivery systems are associated with several advantages and disadvantages such as non-viral vectors being safe, however, they have a low delivery efficiency. Viral vectors, on the other hand, have a greater transfection efficiency but encounter the issues of being cytotoxic and immunogenic. To reduce toxicity and improve transfection efficiency, chemical modifications and conjugations are being developed (Dasgupta and Chatterjee 2021). Thus, there is a need to design safe and effective future delivery systems which could combine the benefits of both delivery systems to employ more miRNA-based therapies from bench to bedside (Yang 2015). In addition to, magnetic particles, along with magnetofection technology, improved the delivery of oligonucleotides (Scherer et al., 2002; Krötz et al., 2003). Moreover, special devices, stereotaxic surgery can be employed for the local delivery of small therapeutic RNA to bypass the blood-brain barrier. Many clinical trials are now investigating MRI-guided focused ultrasound, and demonstrating reversible and safe opening of the blood-brain barrier. Where enhanced delivery of ASOs to the target brain region was achieved by focused ultrasound. Additionally, the safety and cost of oligonucleotide-based therapeutics are yet another key issue commonly faced by these therapeutics. Certain advancement measures such as the combination of oligonucleotide therapeutics with novel delivery modalities enhanced the efficacy and/or improved targeted delivery leading to dose reduction of the drug, which might ultimately reduce costs. One approach to addressing these challenges could be the utilization of computer modeling and artificial intelligence (Roberts et al., 2020). The stability rate of these therapeutics can be enhanced by a series of chemical modifications on the ODN, making them resistant to nucleases. These modifications can be applied to the base, sugar, and phosphodiester group (González Ferreiro et al., 2003). The substitution of the nonbridging oxygen atom in the phosphodiester backbone with a sulphur atom results in a phosphorothioate linkage with improved degradation resistance. Additionally, at the 2′-position, alterations include replacing the hydroxyl group with an O-methoxyethyl (MOE), O-methyl (OMe), amino (NH2) moiety, or fluoro (F), or connecting the 2′ oxygen to the 4′ carbon to produce locked nucleic acid (LNA). These chemical alterations boost the binding affinity of ASOs, siRNAs, aptamers, or other nucleic acids as well as impart enhanced nuclease resistance (Khvorova and Watts 2017). Alterations on both sugar and backbone such as peptide nucleic acid (PNA) and phosphorodiamidate morpholino oligonucleotide (PMO) have also been developed to increase oligomer stability. Altritol nucleic acid, twisted intercalating nucleic acid, cyclohexeny nucleic acid, and hexitol nucleic acid are examples of more recent alterations that have been demonstrated to have higher exon skipping efficiency than 2-OMe ASO *in vitro*. However, the toxicity and the *in vivo* safety profile of these chemistries are still unknown. In fact, the majority of chemical alterations introduced into oligonucleotides have severe toxic consequences. For example, the accumulation of the LNAs within proximal tubule lysosomes is more likely to cause renal toxicity, as shown by tubular necrosis and oligomer accumulation in the kidney biopsy from the first human trial of an LNA ASO (Li et al., 2020). The next major challenge faced is difficulty in the identification of clinically important miRNAs associated with the disease. This is majorly because of the lack of scientific investigation based on miRNAs associated with PD but this is not the only reason it is accompanied by the inconsistency of results in miRNA studies, smaller sample size, different experimental conditions and study designs, use of different PD models, use of several techniques for characterization of miRNA having varying degree of sensitivities, lack of standard protocols for miRNA detection and isolation all these has a negative impact on results. Overall, these factors result in the generation of disagreeing results among various studies performed (Goh et al., 2019).

siRNA-based therapeutics also act as a potential therapeutic tool for several neurological disorders such as AD, PD, etc. Unfortunately, while targeting the brain these therapeutics unlike others have to overcome a lot of *in vivo* obstacles such as systemic degradation, trapping of endosomes, and the blood-brain barrier. With an aim to overcome these flaws several researchers are contributing towards it, which lead to the emergence of several delivery strategies amongst them bioconjugation is one of the potential therapeutic approaches where siRNAs are covalently attached to several biomolecules such as (Polymers, lipids, ligands, antibodies, etc) (Singh et al., 2022). Recently, a study revealed another promising approach for the noninvasive delivery of viral vectors using magnetic resonance-guided focused ultrasound for targeting α-synuclein to multiple brain areas. Thus, improving early diagnosis of PD (Xhima et al., 2018). shRNA also has the potential to suppress γ-synuclein which can be explored as a novel approach (Sun et al., 2020). The next major concern about siRNAs is due to miRNA-like effects their off-target effects leading to unintended gene silencing of up to 1,000 gene transcripts. Thus, it is recommended to maintain the siRNAs level sufficient enough to show just on-target effects. Additionally, chemical modifications such as LNA are needed but their safety profile is yet another challenge for their future application. More researchers are working on devising novel strategies to decrease off-target effects and enhance siRNA specificity (Li, H et al., 2020).

## 10 Conclusion

The introduction to ASOs and miRNAs marks a turning point as a theragnostic approach for various neurological diseases. They have the potential to rectify gene expression, which is at the heart of all treatments, to the extent that this is now achievable. Upon undergoing the process of literature analysis, it is clear that there are various preclinical data available showing the effectiveness of these therapies. But there are several limitations associated with these therapeutics due to limited knowledge of their safety profile as only a few oligonucleotides have completed Phase III clinical studies. As more studies are conducted new side effects begin to unfold such as severe thrombocytopenia or peripheral neuropathy. All drug developers must develop preclinical and clinical safety databases in-order to impart a reasonable degree of confidence which will help the future development of effective oligonucleotide-based drugs for various diseases that currently have limited therapeutic options. The high cost of these therapeutics is also of major concern as many people who are in dire need may not afford them due to a lack of cost-effectiveness. Along with this, there are some other challenges like the blood-brain barrier, rapid degradation by the nucleus, poor cellular uptake, rapid clearance by the kidney, accumulation in the liver, etc. All challenges are being very well understood and researched to overcome these and emerge as a more effective therapeutic approach for the treatment of various neurological disorders including PD. Keeping these challenges in mind several chemical modifications were carried out and actively investigated to improve drug profile like increasing target specificity, enhancing enzyme stability, reducing immune stimulation, and limiting off-target effects. Thus, in the forthcoming years, there are great chances of optimism and we will witness great advances in ASOs and miRNA-based therapeutics.

## Glossary

AmNA: Amino bridged nucleic acid
ALS: Amyotrophic lateral sclerosis
ASOs: Antisense oligonucleotides
BBB: Blood-brain barrier
BMEVs: Bovine milk-derived EVs
DLB: Dementia with Lewy bodies
DA: Dopaminergic neurons
DODAP: 1,2-dioleyl-3-dimethylammonium propane
PD: Parkinson’s disease
LN: Lewy neurites
LB: Lewy bodies
α-syn: α-synuclein
IDPs: Intrinsically disordered proteins
NAC: Non-amyloid-component
β-syn: β-synuclein
miRNA: microRNA
mRNA: Messenger RNA
LNA: Locked nucleic acid
GLUT1: glucose transporter-1
HD: Huntington’s disease
hPSC: Human pluripotent stem cell
siRNA: Small interfering RNA
SMN2: Survival motor neuron
NT: Neurotensin
ICV: Intracerebroventricular injection
Pre-mRNA: Premature mRNA
RNAi: RNA interference
NDD: Novel drug delivery
LNP: Lipid nanoparticle
PEG: Polyethylene glycol
ODN: Oligodeoxynucleotides
PNA: Peptide nucleic acid
PMO: Phosphorodiamidate morpholino oligonucleotide
IND: Indatraline hydrochloride
qPCR: Quantitative polymerase chain reaction
HEK293: Human embryonic kidney 293
PFF: Preformed α-syn fibrils
CTL: Control
PBS: Phosphate buffer solution
MSCs: Mesenchymal stem cells
ERK: Extracellular signal-regulated kinase
SNpc: The pars compacta (SNpc) is a portion of the substantia nigra
SN: Substantia nigra
VTA: ventral tegmental area
LC: Locus coeruleus
NHPs: Non-human primates
ERK: Extracellular signal-regulated kinase
PFF: Preformed fibrils
NLRP3: NLR family pyrin domain containing 3
MEF2D: Myocyte enhancer factor 2D
NFAT5: Nuclear factor of activated T cells 5
snoRNA: Small nucleolar RNA
